# Recombinant human thrombopoietin promotes platelet recovery in DCAG-treated patients with intermediate-high-risk MDS/hypoproliferative AML

**DOI:** 10.1097/MD.0000000000033373

**Published:** 2023-03-31

**Authors:** Xiangli Chen, Yacai Wang, Yuzhu Zang, Zhenghong Wei, Wenhui Zhang, Xiuli Wei, Guangli Luo, Li Chen, Yin Zhang, Zhiwei Xu

**Affiliations:** a Department of Clinical Hematology, Henan Provincial People’s Hospital, People’s Hospital of Zhengzhou University, People’s Hospital of Henan University, Zhengzhou, China; b People’s Hospital of Henan University, Zhengzhou, China; c Department of Hematology, Xinxiang First People’s Hospital, Xinxiang, China; d Department of Hematology, Luohe Central Hospital, Luohe, China; e Department of Hematology, Huaihe hospital, Henan University, Kaifeng, China; f Department of Research Service, Henan Provincial People’s Hospital, People’s Hospital of Zhengzhou University, People’s Hospital of Henan University, Zhengzhou, China.

**Keywords:** acute myeloid leukemia, decitabine, myelodysplastic syndrome, recombinant human thrombopoietin, thrombocytopenia

## Abstract

This study aimed to explore the effects of recombinant human thrombopoietin (rhTPO) on platelet recovery in decitabine, cytarabine, aclarubicin, and G-CSF (DCAG)-treated patients with intermediate-high-risk myelodysplastic syndrome/hypo proliferative acute myeloid leukemia.

**Methods:**

Recruited patients were at a ratio of 1:1 into 2 groups: the rhTPO group (DCAG + rhTPO) and control group (DCAG). The primary endpoint was the time for platelets to recover to ≥ 20 × 10^9^/L. The secondary endpoints were the time for platelets to recover to ≥ 30 × 10^9^/L and ≥ 50 × 10^9^/L, overall survival (OS), and progression-free survival (PFS).

**Results:**

The time required for platelet recovery to ≥ 20 × 10^9^/L, ≥30 × 10^9^/L, and ≥ 50 × 10^9^/L in the rhTPO group was significantly shorter (6.5 ± 2.2 vs 8.4 ± 3.1 days, 9.0 ± 2.7 vs 12.2 ± 3.9 days, 12.4 ± 4.7 vs 15.5 ± 9.3 days, respectively; all *P* < .05 vs controls). The amount of platelet transfusion in the rhTPO group was smaller (4.4 ± 3.1 vs 6.1 ± 4.0 U, *P* = .047 vs controls). The bleeding score was lower (*P* = .045 vs controls). The OS and PFS were significantly different (*P* = .009 and *P* = .004). The multivariable analysis showed that age, karyotype, and time for PLT recovery to ≥ 20 × 10^9^/L were independently associated with OS. Adverse events were similar.

**Conclusions:**

This study suggests that rhTPO leads to a faster platelet recovery after DCAG treatment, reduces the risk of bleeding, reduces the number of platelet transfusions, and prolongs the OS and PFS.

## 1. Introduction

Myelodysplastic syndrome (MDS) is a heterogeneous group of clonal disorders characterized by ineffective hematopoiesis leading to peripheral blood cytopenias and transformation to acute myelogenous leukemia (AML).^[1–3]^ MDS is characterized by ineffective hematopoiesis, dysplasia, peripheral blood cell reduction, progressive bone marrow failure, and high risk of transformation to AML. AML is a collection of heterogeneous hematopoietic stem cell disorders characterized by incomplete maturation of blood cells and reduced production of normal hematopoietic elements.^[4,5]^The occurrence of thrombocytopenia in patients with MDS is as high as 40% to 65%,^[6]^ and 14% to 30% of patients directly die from bleeding caused by thrombocytopenia.^[7]^ In 2012, thrombocytopenia was included in the revised International Prognostic Scoring System (IPSS-R) as an independent predictor of poor prognosis for MDS.^[8]^

Decitabine, a hypomethylating agent that inhibits DNA methyltransferase, has been approved for the treatment of MDS/AML patients. Study has shown that the effects of decitabine in thrombocytopenia patients may be due to enhanced megakaryocyte differentiation and maturation.^[9]^ The decitabine, cytarabine, aclarubicin, and G-CSF (DCAG) regimen (decitabine plus cytarabine, low-dose aclarubicin, and granulocyte colony-stimulating factor priming) achieves a definite clinical efficacy in the treatment of intermediate-high-risk MDS and hypo proliferative or senile AML,^[1,5,10]^ but the main side effect of this regimen is severe, long-term thrombocytopenia after chemotherapy. A survey in the United States showed that 6% to 33% of patients with MDS have to rely on platelet transfusion, with all the inherent risks.^[11]^

Thrombopoietin (TPO), a naturally occurring, glycosylated polypeptide that is cloned by Genentech in 1994, is capable of inducing differentiation of stem cells into megakaryocytes and accelerating the maturation of megakaryocytes, thereby increasing the platelet count. TPO mainly comes from liver, kidney and bone marrow stromal cells; It is an endogenous growth factor that activates MAP kinase, JAK/ STAT by binding to specific receptor c-MPL; they are involved in regulating the production and differentiation of megakaryocytes and platelets in bone marrow, thereby increasing the number of platelets.^[12]^ Recombinant human thrombopoietin (rhTPO) can promote bone marrow cells of MDS patients to differentiate into megakaryocyte lineage, rhTPO has good tolerance, is very effective in improving platelet count, and can protect megakaryocytes from chemotherapy-induced apoptosis.^[13]^ The combination of decitabine and rhTPO may synergetic promote the differentiation and maturation of megakaryocytes and accelerate the recovery of platelets.

The clinical application of TPO in the treatment of primary immune thrombocytopenic purpura (ITP) achieved a definite therapeutic effect and had become the first-line recommended treatment.^[14]^ The most representative platelet growth-promoting factor available in China is rhTPO. rhTPO had been approved in China for patients with chronic ITP with failure to glucocorticoid therapy and achieved a high response rate of 60%.^[15]^

Nevertheless, whether rhTPO can be used in patients with intermediate-high-risk MDS/hypo proliferative AML has not been verified. Therefore, this study aimed to explore the therapeutic effect of rhTPO on thrombocytopenia and prognosis of patients with intermediate-high-risk MDS/hypo proliferative AML who were treated with the DCAG regimen.

## 2. Methods

### 2.1. Study design and patients

This was a multicenter randomized controlled open label clinical trial of patients with intermediate-high-risk MDS/hypo proliferative AML and treated with the DCAG regimen from May 31, 2018, to October 31, 2020. The centers included: Henan Provincial People’s Hospital, Luoyang Central Hospital Affiliated to Zhengzhou University, Luohe Central Hospital, Xinxiang First People’s Hospital, and the First Affiliated Hospital of Henan University of Science and Technology. The trial was approved by the ethics committee of the Henan Province People’s Hospital [2019 (54)]. All patients signed the informed consent form prior to any study procedure. The trial was performed according to the tenets of the Declaration of Helsinki and to the Good Clinical Practice. The trial was registered (#ChiCTR2000038982).

The inclusion criteria were: 14 to 70 years of age (adolescents 14–18 years of age had to be at least 150-cm tall and weigh at least 50 kg); Diagnosis of MDS with an excess of blasts (MDS-EB-I, MDS-EB-II), or hypo proliferative AML transformed from MDS^[5,16]^; Eastern Cooperative Oncology Group (ECOG) score 0 to 2; First chemotherapy ever, or the interval between the last chemotherapy and this study was ≥ 3 months if previous chemotherapy was ineffective or effective but relapsed; Expected survival ≥ 3 months; No severe heart, lung, liver, and kidney disease; and Able to understand the trial and willing to sign the informed consent form.

The exclusion criteria were: Previous allergies to drugs included in the test protocol or drugs with similar chemical structure to the tested drug; Pregnant and lactating women and women of childbearing age who were unwilling to take contraception; Active infection; With extramedullary lesions; Drug addiction or long-term alcohol abuse that could affect the evaluation of the test results; Unable to obtain informed consent due to mental disorders or other conditions or unable to cooperate with the completion of the experimental treatment and examination; Clinically significant QTc interval extension (male > 450 ms, female > 470 ms), ventricular tachycardia, atrial fibrillation, cardiac conduction block above II degree, myocardial infarction within 1 year, congestive heart failure, or coronary heart disease with symptoms that required medical treatment; Abnormal liver function (total bilirubin > 1.5 times the upper limit of normal value (ULN), ALT/AST > 2.5 times the ULN or ALT/AST > 5 times the ULN in patients with liver invasion), abnormal renal function (serum creatinine > 1.5 times the ULN); or Patients who were judged by the investigators as being not suitable to participate in this trial.

The efficacy evaluation standard MDS was evaluated according to the International Working Group efficacy evaluation standard revised in 2006, and the efficacy evaluation standard of AML was based on the 2017 European Leukemia Net efficacy evaluation standard.

### 2.2. Randomization and blinding

Randomization was performed using a random number table that was generated on May 7, 2018. The patients were sequentially included and assigned the next random number. Patients with odd numbers were assigned to the rhTPO group, and the patients with even numbers were assigned to the control group. In the case of uneven group size, assignation numbers from the larger groups were randomly selected to complete the smaller group. Group assignation was performed by contacting the biostatistician at the lead center. The patients and physicians were not blind to grouping. Only the statisticians were blinded.

### 2.3. Intervention

Both groups were treated with DCAG chemotherapy: decitabine (10 mg/d, d1–d10); cytarabine (15 mg/m^2^, q12 hours, d1–14); aclarubicin (20 mg/day, qod, d1–10); and granulocyte stimulating factor (150µg/m^2^, q12 hours, d0–14). During chemotherapy, symptomatic treatments such as stomach protection, antiemetic, heart protection, liver function protection, hydration, and alkalization were routinely performed. Each patient underwent 2 courses of treatment.

In the rhTPO group, rhTPO (Terbiao, Shenyang Sansheng Pharmaceutical Co., Ltd., Shenyang, China) was used when the platelets were < 30 × 10^9^/L. The dose was 300 µg/kg. It was discontinued when the platelets were > 50 × 10^9^/L. rhTPO was not used in the control group. The patients in the 2 groups were given platelet transfusion when the platelets were < 20 × 10^9^/L or had obvious bleeding symptoms, and etamsylate (dicynone) injection 0.5 g tid was used. Except rhTPO, no other drugs, such as romiplostim and eltrombopag, were used to promote platelet recovery with all patients.

### 2.4. Follow up

The liver, kidney, and blood coagulation functions were examined before each chemotherapy cycle. From the first day of chemotherapy, body temperature and feces and urine status were monitored. Blood routine was examined every other day to monitor the count of the 3 series of cells.

After completing 2 courses of DCAG, the patients were selectively treated with IA (idarubicin 10 mg/m^2^, d1–3, cytarabine 150 mg/m^2^, d1–7), DCAG, medium dose cytarabine (2000 mg/m^2^, d1–5) and other schemes for consolidation and maintenance, and some patients underwent stem cell transplantation. Follow up ended on October 31, 2021.

### 2.5. Endpoints

The primary endpoint was the time for platelets to recover to ≥ 20 × 10^9^/L. when platelets begin to rise to 20 × 10^9^/L and platelets are not infused within 3 days, platelets can stabilize at 20 × 10^9^/L or above, then the time when platelets reach 20 × 10^9^/L for the first time is the cutoff point. The secondary endpoints were the time for platelets to recover to ≥ 30 × 10^9^/L, ≥50 × 10^9^/L, and ≥ 100 × 10^9^/L, bleeding score (according to the ITP bleeding grading system 2016 version in Table S1, Supplemental Digital Content, http://links.lww.com/MD/I714), amount of platelet transfusion, hospitalization time, overall survival (OS), and progression-free survival (PFS). OS was defined as the time from the enrollment experiment to death (from any cause) or to the end of follow up. PFS was defined as the time from the enrollment experiment to the observation of disease progression or death. adverse events (AEs) like bleeding, rash, mucositis, and cardiotoxicity were observed. Nausea and vomiting and other gastrointestinal reactions during chemotherapy were investigated. The pulmonary infection and perianal infection were closely screened for during observation.

### 2.6. Sample size calculation

rhTPO was first used preliminary in 20 patients. The result revealed that the use of rhTPO significantly promoted platelet recovery, shortened hospital stay, and reduced the amount of platelet transfusion (compared with historical controls). Therefore, considering a difference in the time of platelets being ≥ 20 × 10^9^/L between the 2 groups of 2.0, α = 0.05, a power of 90%, and a lost to-follow up rate of about 20%, the sample size was estimated to be at least 68 cases or at least 34/group. As per the study protocol, 50 patients were recruited in each group.

### 2.7. Statistical analysis

SPSS 22.0 (IBM, Armonk, NY) was used for data processing. Continuous variables that conformed to the normal distribution (Kolmogorov-Smirnov test) were presented using means ± standard deviations and were analyzed using Student *t* test; otherwise, they were presented using medians (ranges) and compared using the Mann-Whitney *U* test. Categorical variables were presented as frequencies (%) and analyzed using the chi-square test. The OS and PFS were presented using the Kaplan–Meier method and analyzed using the log-rank. Univariable and multivariable and Cox regression analyses were used to evaluate the factors associated with OS. Factors with *P* < .10 in the univariable analyses were included in the multivariable Cox regression model. Two-sided (except for the chi-square test) *P* values < .05 were considered statistically significant.

## 3. Results

### 3.1. Inclusion and exclusion of the participants

A total of 126 patients met the inclusion criteria, and 14 were excluded (5 patients had lung infections before enrollment, 3 had family members who did not agree with participation, 3 had arrhythmia according to ECG, one had a long-term alcohol abuse, and 2 had increased transaminases without obvious reasons). Therefore, 112 patients were randomized, with 56 cases in each group. Two patients in the rhTPO group were not readmitted after completing a course of treatment and were lost to follow up early. Four patients in the rhTPO group and 6 in the control group withdrew from the trial. Eventually, 100 participants were included in the analysis, with 50 cases in each group. Among them, 2 participants in the rhTPO group and 3 in the control group were loss of follow up in the late stage, but their follow up was over 6 months, and they were included in the analysis (Figure S1, Supplemental Digital Content, http://links.lww.com/MD/I715).

### 3.2. Characteristics of the participants

Table 1 presents the characteristics of the participants. The median age was 53 years (range, 15–70 years). There were 62 males and 38 females. Forty-six participants had comorbidities. There were 28 participants with hypo proliferative AML, and 72 with MDS. According to the WHO classification criteria (2016), there were 31 patients with MDS-EB-1 (which included 5 patients with bone marrow [BM] blast cells < 5%, but which blast cells in the peripheral blood were 2%–4%.) and 41 with MDS-EB-2. For the IPSS-R of the 72 participants with MDS, there were 13 with moderate risk, 39 with high risk, and 20 with extremely high risk. There were no significant statistical differences between the 2 groups in terms of age, sex, presence of comorbidities, white blood cells, hemoglobin, platelets, chemotherapy prior to recruitment, disease type, IPSS-R score, bone marrow blast cell number, number of series with dyshematopoiesis, and chromosome karyotype at initial diagnosis (all *P* > .05).

**Table 1:** Characteristics of the participants.

	rhTPO (n = 50)	Control (n = 50)	*P* value
Age (yr)	53.5 ± 13.3	49.4 ± 14.1	.184
Sex (male)	32 (64.0%)	30 (60.0%)	.680
With comorbidities	22 (44.0%)	24 (48.0%)	.688
White blood cells (×10^9^/L)	6.1 ± 6.8	6.6 ± 6.9	.745
Hemoglobin (g/L)	77.5 ± 20.8	75.7 ± 21.2	.707
Platelets (×10^9^/L)	82.5 ± 55.2	72.3 ± 50.9	.394
Chemotherapy prior to recruitment			.230
No	29 (58.0%)	23 (46.0%)	
Yes	21 (42.0%)	27 (54.0%)	
Disease type			.358
AML	12 (24.0%)	16 (32.0%)	
MDS-EB-I	14 (28.0%)	17 (34.0%)	
MDS-EB-II	24 (48.0%)	17 (34.0%)	
IPSS-R grouping			.240
Intermediate risk	9 (23.7%)	4 (11.8%)	
High risk	17 (44.7%)	22 (64.7%)	
Extremely high risk	12 (31.6%)	8 (23.5%)	
Number of bone marrow primitive cells			.447
<5%	3 (6.0%)	2 (4.0%)	
≥5% and < 10%	11 (22.0%)	15 (30.0%)	
≥10% and < 20%	24 (48.0%)	17 (34.0%)	
≥20%	12 (24.0%)	16 (32.0%)	
Dyshematopoiesis			.471
0–1 lineages	16 (32.0%)	15 (30.0%)	
2 lineages	9 (18.0%)	14 (28.0%)	
3 lineages	25 (50.0%)	21 (42.0%)	
Karyotype			.257
Good	23 (46.0%)	15 (30.0%)	
Moderate	18 (36.0%)	25 (50.0%)	
Poor	9 (18.0%)	10 (20.0%)	

Comorbidities included diabetes, hypertension, and chronic hepatitis B.

Karyotype: good: normal karyotype, -Y, del(20q), del(5q); poor: complex (≥3 abnormalities) or abnormal chromosome 7; moderate: other abnormalities.

Number of bone marrow primitive cells < 5%.

AML = acute myeloid leukemia, IPSS-R = the revised International Prognostic Scoring System, MDS-EB = MDS with an excess of blasts, rhTPO = recombinant human thrombopoietin.

### 3.3. Primary endpoint

The time for platelet recovery to ≥ 20 × 10^9^/L after chemotherapy in the rhTPO group was significantly shorter than the control group (6.5 ± 2.2 vs 8.4 ± 3.1 days, *P* = .003) (Table 2).

**Table 2:** The time for platelets to recover to ≥ 20, 30, 50, and 100 × 10^9^/L.

	rhTPO	Control	*P* value
Platelets ≥ 20 × 10^9^/L (days)	n = 50	n = 50	
	6.5 ± 2.2	8.4 ± 3.1	.003
Platelets ≥ 30 × 10^9^/L (days)	n = 50	n = 50	
	9.0 ± 2.7	12.2 ± 3.9	.032
Platelets ≥ 50 × 10^9^/L (days)	n = 38	n = 33	
	12.4 ± 4.7	15.5 ± 9.3	.036
Platelets ≥ 100 × 10^9^/L (days)	n = 26	n = 22	
	20.8 ± 11.2	23.0 ± 19.8	.069

rhTPO = recombinant human thrombopoietin.

### 3.4. Secondary endpoints

The time for platelet recovery to ≥ 30 × 10^9^/L and ≥ 50 × 10^9^/L after chemotherapy in the rhTPO group was both shorter than the control group (9.0 ± 2.7 vs 12.2 ± 3.9 days, *P* = .032; 12.4 ± 4.7 vs 15.5 ± 9.3 days, *P* = .036). There were no stat istically significant differences in the time for platelet recovery to ≥ 100 × 10^9^/L between the 2 groups (*P* = .069) (Table 2). The amount of platelet transfusion in the rhTPO group was less than that of the control group (4.4 ± 3.1 vs 6.1 ± 4.0 U, *P* = .047). The hospitalization time of the rhTPO group to complete each course of treatment was shorter than that of the control group (22.0 ± 5.9 vs 28.5 ± 7.7 days, *P* = .043). There were no severe visceral and central nervous system bleeding in both groups of patients. The numbers of patients with bleeding scores of 0, 1, 2, and 3 points in the rhTPO group were 37, 8, 4, and 1, respectively. The numbers of patients with bleeding scores of 0, 1, 2, and 3 points in the control group were 27, 8, 7, and 8, respectively. The difference in the distribution of the bleeding scores between the 2 groups was statistically significant (*P = *.045) (Table S2, Supplemental Digital Content, http://links.lww.com/MD/I716).

### 3.5. Treatment response

All 100 patients received 2 cycles of DCAG chemotherapy. In the rhTPO group, there were 6 patients (12.0%) with complete remission (CR), 27 (54.0%) with marrow complete remission (mCR); 5 (10.0%) with partial remission (PR), 4 (8.0%) with hematological improvement (HI), and 8 (16.0%) with non-remission. The overall response rate (ORR; CR + mCR + PR + HI) was 84% in the rhTPO group. In the control group, there were 4 patients (8.0%) with CR, 29 (58.0%) with mCR, 2 (4.0%) with PR, 5 (10.0%) with HI, and 10 (20.0%) with non-remission. The ORR was 80%. Both groups of patients obtained a relatively high ORR, with no significant statistical significance between the 2 groups (*P* = .466) (Table S3, Supplemental Digital Content, http://links.lww.com/MD/I717).

### 3.6. AEs

The main AEs during treatment were bone marrow suppression and infection. All patients had grade III to IV bone marrow suppression. In the rhTPO group, 16 patients (32.0%) had infections, including 11 (22.0%) with lung infection, 1 (2.0%) with oral infection, 2 (4.0%) with bacteremia, and 2 (4.0%) with upper respiratory tract infection. In the control group, 14 (28.0%) had infections, including 8 (16.0%) with lung infection, 1 (2.0%) with oral infection, 2 (4.0%) with bacteremia, 2 (4.0%) with perianal infection, and 1 (2.0%) with urinary tract infection. There was no statistically significant difference in the occurrence rates of infection between the 2 groups (32.0% vs 28.0%, *P* = .663). There were 4 patients (8.0%) with significant liver function damage (transaminases increased more than twice the ULN) in the rhTPO group, and 3 (6.0%) in the control group (*P = *.695). Fifteen participants (30.0%) in the rhTPO group and 17 (34.0%) in the control group had intestinal reactions (*P* = .669) (Table S4, Supplemental Digital Content, http://links.lww.com/MD/I718). In addition, no obvious thrombosis and leukemia transformation were found in both groups.

### 3.7. Survival

**Figure 1.**
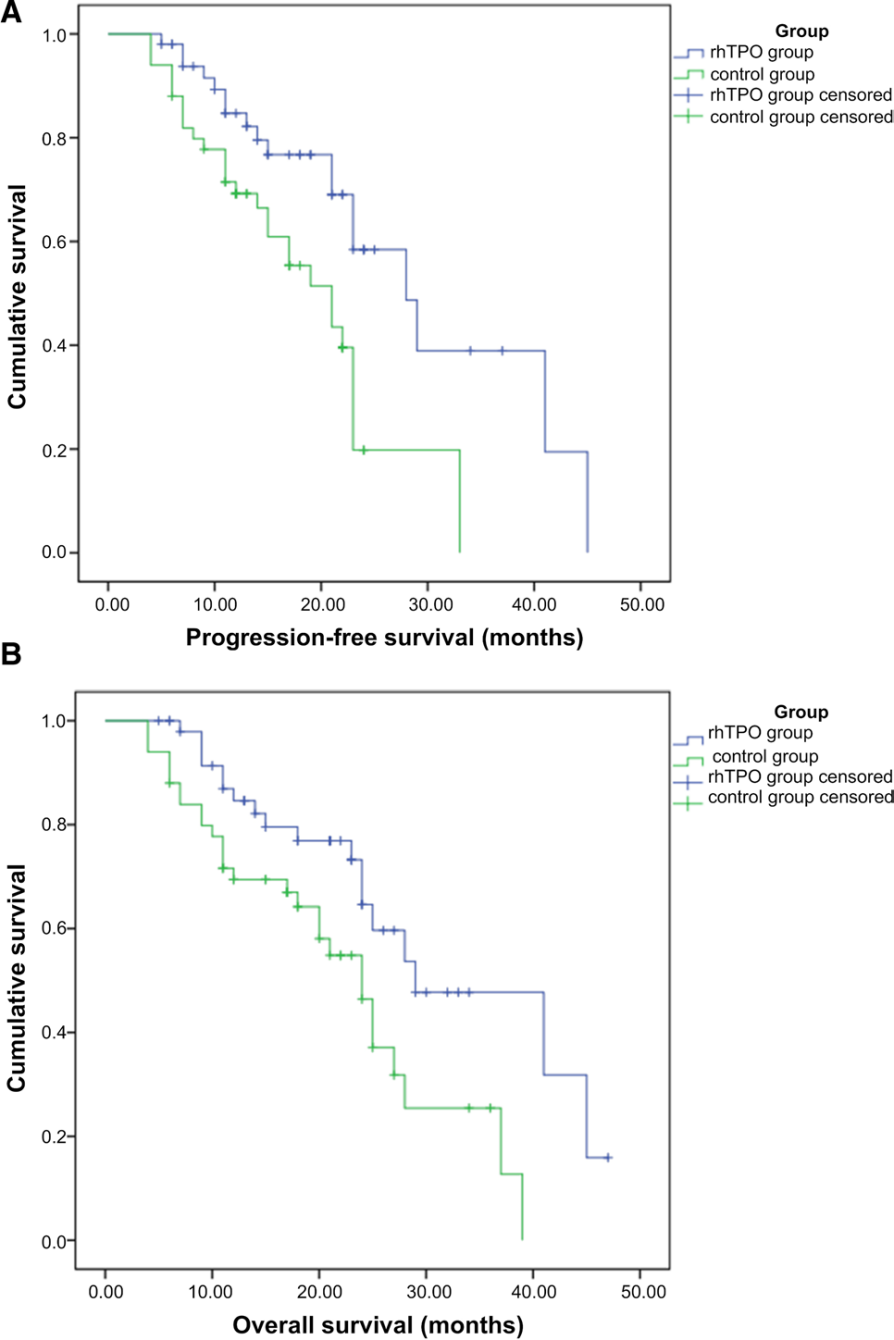
(A) OS analysis of rhTPO group and control group. The overall survival (OS) in the rhTPO group was significantly longer than the control group (*P* = .009). (B) PFS analysis of rhTPO group and control group. The progression-free survival (PFS) in the rhTPO group was significantly longer than the control group (*P* = .004). rhTPO = recombinant human thrombopoietin.

As of October 31, 2021, 18 participants had died in the rhTPO group due to disease progression, 2 were lost to follow-up in the late stage, and 30 were alive. In the control group, 28 participants had died, 3 were lost for follow-up in the late stage, and 19 were alive. The median OS in the rhTPO group was 29 months (95% CI: 17.137–40.863), compared with 24 months (95% CI: 19.973–28.027) in the control group (*P* = .009) (Fig. 1A). The median PFS of rhTPO group was 28 months (95% CI: 20.790–35.210), compared with 21 months (95% CI: 15.348–26.652) in the control group (*P* = .004) (Fig. 1B).

### 3.8. Factors associated with OS

Significant differences (*P* < .10) were observed in the univariable analyses for age, disease type, bone marrow blast cell number, karyotype, time to platelet recovery to ≥ 20 × 10^9^/L, and bleeding (Table 3). The Cox multivariable regression model showed that age (OR = 2.46, 95% CI: 1.04–5.84, *P* = .042), karyotype (OR = 1.57, 95% CI: 1.04–2.38, *P* = .033), and time for platelet recovery to ≥ 20 × 10^9^/L (OR = 2.20, 95% CI: 1.16–4.20, *P* = .016) were independently associated with OS (Table 4).

**Table 3:** Univariable analysis of the factors affecting the OS (log-rank).

Factors		N	Median OS (mo)	95% CI	*P* value
Age (yr)	<65	83	23	21.955–24.045	.091
	≥65	17	21	6.326–35.674	
Sex	Male	62	23	21.176–24.824	.526
	Female	38	22	14.480–29.520	
Disease type	MDS-EB-I	31	45	--	.040
	MDS-EB-II	41	23	20.226–25.774	
	AML	28	17	9.165–24.835	
Chemotherapy prior to recruitment	No	52	27	22.788–31.212	.603
	Yes	48	25	20.769–29.231	
Number of bone marrow primitive cells	<5%	5	21	14.599–27.401	.062
	≥5% and < 10%	26	45	--	
	≥10% and < 20%	41	23	17.959–28.041	
	≥20%	28	21	14.471–27.529	
Dyshematopoiesis	0–1 series	31	23	19.956–26.044	.369
	2 series	23	29	16.371–41.609	
	3 series	46	28	17.661–38.339	
Karyotype	Good	38	28	16.398–39.604	.027
	Moderate	43	21	15.761–26.239	
	Poor	19	21	10.712–31.288	
Platelets ≥ 20 × 10^9^/L (d)	≤7	69	27	23.599–30.401	.080
	>7	31	24	17.081–30.919	
Platelets ≥ 30 × 10^9^/L (d)	≤10	59	27	24.122–29.878	.593
	>10	41	25	18.041–31.959	
Bleeding	Yes	23	24	16.830–31.170	.079
	No	77	29	18.037–39.963	

AML = acute myeloid leukemia, CI = confidence interval, MDS-EB = MDS with an excess of blasts, OS = overall survival.

**Table 4:** Multivariable Cox regression of factors affecting the OS of patients.

Factors	*P* value	OR	95% CI
Age	.042	2.458	1.035–5.841
Karyotype	.033	1.573	1.038–2.384
Platelets ≥ 20 × 10^9^/L (d)	.016	2.203	1.157–4.197

CI = confidence interval, OR = odds ratio, OS = overall survival.

## 4. Discussion

The DCAG regimen is effective for intermediate-high-risk MDS/hypo proliferative AML, but the thrombocytopenia is a major adverse effect. This multicenter randomized controlled trial aimed to explore the effects of rhTPO in such patients. This study results show that the time for platelet recovery to ≥ 20 × 10^9^/L, ≥30 × 10^9^/L, and ≥ 50 × 10^9^/L was faster in the rhTPO group compared with controls. The multivariable analysis showed that the time for platelets recovery to ≥ 20 × 10^9^/L was an independent factor influence OS. In addition, rhTPO shortened hospitalization time, reduced the amount of platelet transfusion, and also reduced the patient’s risk of bleeding. Therefore, this study suggests that rhTPO leads to a faster platelet recovery after DCAG treatment, reduces the risk of bleeding, reduces the number of platelet transfusions, and prolongs the OS and PFS.

Song et al^[17]^ studied patients with severe aplastic anemia after allogeneic hematopoietic stem cell transplantation and observed that the time for platelet recovery to ≥ 50 × 10^9^/L in the rhTPO group was significantly shorter than that of the control group, which is consistent with our study in MDS/AML. On the other hand, they observed no significant difference for the time of platelet recovery to ≥ 20 × 10^9^/L, which was different from the present study, and might be because the type of disease treated and the state of the disease are different. The pretreatment plan was to destroy the patient’s original hematopoietic stem cells first and inject the donor hematopoietic stem cells into the patient’s body. The proliferation, differentiation, and maturation took a relatively long time. In addition, the number of megakaryocytes in the early stage after transplantation was relatively small. Sun et al^[18]^ observed that the rate of platelet restores to ≥ 20 × 10^9^ was higher with rhTPO than in historical controls after allogeneic stem cell transplantation.

The results of this study also showed that when the platelet levels were similar, the rhTPO group had a lower risk of bleeding. The bleeding score of the rhTPO group was significantly lower than that of the control group, which was consistent with clinical symptoms. The amount of platelet transfusion of patients in the rhTPO group is significantly lower than that of the control group. Tang Guang et al^[19]^ found that rhTPO can shorten the duration of thrombocytopenia and the amount of platelet transfusion after chemotherapy for acute myeloid leukemia, promote platelet recovery, reduce the risk of bleeding, reduce the occurrence of adverse reactions, and is well tolerated. This is consistent with our results.

Wang et al^[20]^ analyzed 275 patients who underwent allogeneic hematopoietic stem cell transplantation. Their multivariable analysis showed that platelet implantation dysfunction was associated with the absence of rhTPO; further analysis showed that rhTPO could significantly improve OS in patients with MDS and AA (*P = *.014). In the present study, The rhTPO improved the OS and PFS of the rhTPO group. Liu YM, et al^[21]^ reported a novel function of rhTPO in increasing hematopoietic stem and progenitor cell (HSPC) homing to the BM. Single doses of TPO treatment to the recipients immediately after BM transplantation showed significantly improved homing of HSPCs to the BM, which subsequently resulted in enhanced short- and long-term engraftment of HSPCs in mice. The application of rhTPO could promote the platelet recovery as soon as possible and shorten the hospitalization time of patients, reduces the risk of bleeding. All of these promoted patients to use chemotherapy drugs more actively, normatively and adequately, which also further improved the survival time of patients.

So far, no studies have shown that rhTPO can promote the proliferation of leukemia cells. But Wang N et al^[22]^ study had shown that rhTPO could not promote the proliferation of Kasumi-1, Skno-1, HEL, HL-60, and THP-1 leukemia cell lines; On the contrary, rhTPO could inhibit HL-60 cell proliferation and promote its apoptosis, and this effect was not related to c-MPL gene expression or protein expression. The other study indicated a transient morphological change that might be similar to that observed in chronic myeloproliferative disorders, but those changes reverse by themselves when rhTPO is stopped.^[23]^ In the present study, during the application of rhTPO combined with DCAG in the treatment of intermediate-high-risk MDS, no conversion from MDS to AML was observed during treatment, which might be because rhTPO was not used alone but combined with DCAG. Further studies with a larger sample size are needed to prove whether rhTPO promote megakaryocyte proliferation while increasing the risk of MDS progressing to AML.

Of course, this study has limitations. The sample size was relatively small and limited to a single ethnic group. The patients and investigators were not blind to grouping. The pharmacokinetic and pharmacodynamic of rhTPO were not examined. Additional studies are still necessary to confirm the benefits and safety of rhTPO for MDS/AML.

In conclusion, the present study strongly suggests that rhTPO leads to a faster platelet recovery after DCAG treatment, reduces the risk of bleeding, reduces the number of platelet transfusions, and prolongs the OS and progression-free survival (PFS).

**Table:** 

	Blast cells in the Bone marrow	Blast cells in the peripheral blood (In the absence of G-CSF, the results of 2 tests)
1	4.8%	2%, 4%
2	1%	3%, 3%
3	3%	3%, 4%
4	4.8%	2%, 3%
5	4.2%	3%, 4%

## Author contributions

**Conceptualization:** Xiangli Chen, Yuzhu Zang.

**Data curation:** Yacai Wang, Zhenghong Wei, Zhiwei Xu.

**Formal analysis:** Xiangli Chen, Yacai Wang.

**Funding acquisition:** Xiangli Chen.

**Methodology:** Zhiwei Xu.

**Project administration:** Xiangli Chen, Yacai Wang, Yin Zhang.

**Resources:** Xiangli Chen, Zhenghong Wei, Wenhui Zhang, Xiuli Wei, Guangli Luo, Li Chen.

**Supervision:** Yin Zhang.

**Writing – original draft:** Xiangli Chen, Yacai Wang, Yuzhu Zang.

**Writing – review & editing:** Xiangli Chen, Yacai Wang, Zhenghong Wei, Wenhui Zhang, Xiuli Wei, Guangli Luo, Li Chen, Yin Zhang, Zhiwei Xu.

## Supplementary Material










