# Supplementary material for: Recombinant human thrombopoietin promotes platelet recovery in DCAG-treated patients with intermediate-high-risk MDS/hypoproliferative AML
Source: Medicine (Baltimore). 2023 Mar 31;102(13):e33373. doi: 10.1097/MD.0000000000033373 (PMC10063278; doi:10.1097/MD.0000000000033373)
Supplement: Supplementary file 1 [file medi-102-e33373-s001.pdf]

Supplementary Table S1. The ITP bleeding grading system (2016 version)

Scores	Age (years)		Dermatorrhagia (petechiae/ecchymosis/hematoma)		Mucosal hemorrhage (nasal gums/oral blood blister conjunctiva)			Deep organ bleeding<br>Viscera (lung, gastrointestinal tract, urogenital system)			Central nervous system
	≥65	≥75	Head and face	Other sites	Occasional, can spontaneously stop.	Multiple bleeding, difficult to be stopped.	Combined with anemia.	Without anemia	With anemia	Life-threatening	
1	✓			✓							
2		✓	✓		✓						
3						✓		✓			
5							✓		✓		
8										✓	✓
