# Supplementary figures and images for: Recombinant human thrombopoietin promotes platelet recovery in DCAG-treated patients with intermediate-high-risk MDS/hypoproliferative AML
Source: Medicine (Baltimore). 2023 Mar 31;102(13):e33373. doi: 10.1097/MD.0000000000033373 (PMC10063278; doi:10.1097/MD.0000000000033373)

Supplementary Figure 1. The Flowchart of this clinical trial

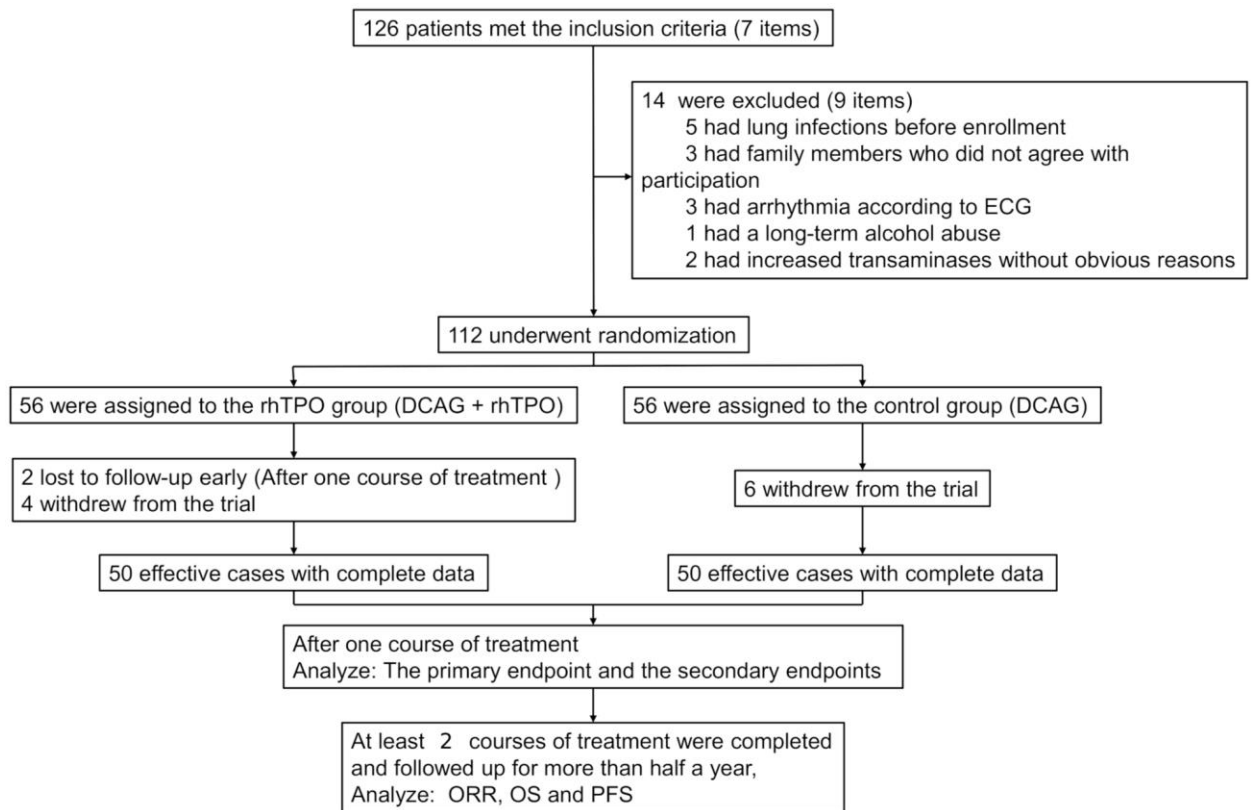

Supplement: Supplementary file 2 [file medi-102-e33373-s002.pdf]
