# Supplementary material for: Recombinant human thrombopoietin promotes platelet recovery in DCAG-treated patients with intermediate-high-risk MDS/hypoproliferative AML
Source: Medicine (Baltimore). 2023 Mar 31;102(13):e33373. doi: 10.1097/MD.0000000000033373 (PMC10063278; doi:10.1097/MD.0000000000033373)
Supplement: Supplementary file 3 [file medi-102-e33373-s003.pdf]

Supplementary Table S2. Platelet transfusion, length of hospital stay, and bleeding scores

	rhTPO (n=50)	Control (n=50)	*P*
Platelet transfusion volume (U)	4.4±3.1	6.1±4.0	0.047
Length of hospital stay (days) *	22.0±5.9	28.5±7.7	0.043
Bleeding scores			0.045
0	37 (74.0%)	27 (54.0%)	
1	8 (16.0%)	8 (16.0%)	
2	4 (8.0%)	7 (14.0%)	
3	1 (2.0%)	8 (16.0%)	

* Some patients who had prolonged hospitalization due to infection were excluded: 11 cases in the rhTPO group and 13 cases in the control group.

rhTPO, recombinant human thrombopoietin.
