# Supplementary material for: Recombinant human thrombopoietin promotes platelet recovery in DCAG-treated patients with intermediate-high-risk MDS/hypoproliferative AML
Source: Medicine (Baltimore). 2023 Mar 31;102(13):e33373. doi: 10.1097/MD.0000000000033373 (PMC10063278; doi:10.1097/MD.0000000000033373)
Supplement: Supplementary file 4 [file medi-102-e33373-s004.pdf]

Supplementary Table S3. Remission rates

	rhTPO (n=50)	Control (n=50)	*P*
Complete remission	6 (12.0%)	4 (8.0%)	0.466
Bone marrow remission	27 (54.0%)	29 (58.0%)	
Partial remission	5 (10.0%)	2 (4.0%)	
Hematological improvement	4 (8.0%)	5 (10.0%)	
Non-remission	8 (16.0%)	10 (20.0%)	

rhTPO, recombinant human thrombopoietin.
