# Supplementary material for: Recombinant human thrombopoietin promotes platelet recovery in DCAG-treated patients with intermediate-high-risk MDS/hypoproliferative AML
Source: Medicine (Baltimore). 2023 Mar 31;102(13):e33373. doi: 10.1097/MD.0000000000033373 (PMC10063278; doi:10.1097/MD.0000000000033373)
Supplement: Supplementary file 5 [file medi-102-e33373-s005.pdf]

Supplementary Table S4. Adverse events

Adverse reactions	rhTPO (n=50)	Control (n=50)	*P*
Myelosuppression (III-IV)	50 (100.0%)	50 (100.0%)	---
Infection	16 (32.0%)	14 (28.0%)	0.663
Liver function damage	4 (8.0%)	3 (6.0%)	0.695
Gastrointestinal reactions	15 (30.0%)	17 (34.0%)	0.669

rhTPO, recombinant human thrombopoietin.
